# Degradation of 3-Phenoxybenzoic Acid by a *Bacillus* sp

**DOI:** 10.1371/journal.pone.0050456

**Published:** 2012-11-30

**Authors:** Shaohua Chen, Wei Hu, Ying Xiao, Yinyue Deng, Jianwen Jia, Meiying Hu

**Affiliations:** 1 Key Laboratory of Pesticide and Chemical Biology, Ministry of Education, and Laboratory of Insect Toxicology, South China Agricultural University, Guangzhou, P. R. China; 2 Institute of Molecular and Cell Biology, Proteos, Singapore, Republic of Singapore; National University of Singapore, Singapore

## Abstract

3-Phenoxybenzoic acid (3-PBA) is of great environmental concern with regards to endocrine disrupting activity and widespread occurrence in water and soil, yet little is known about microbial degradation in contaminated regions. We report here that a new bacterial strain isolated from soil, designated DG-02, was shown to degrade 95.6% of 50 mg·L^−1^ 3-PBA within 72 h in mineral salt medium (MSM). Strain DG-02 was identified as *Bacillus* sp. based on the morphology, physio-biochemical tests and 16S rRNA sequence. The optimum conditions for 3-PBA degradation were determined to be 30.9°C and pH 7.7 using response surface methodology (RSM). The isolate converted 3-PBA to produce 3-(2-methoxyphenoxy) benzoic acid, protocatechuate, phenol, and 3,4-dihydroxy phenol, and subsequently transformed these compounds with a *q*_max_, *K*_s_ and *K*_i_ of 0.8615 h^−1^, 626.7842 mg·L^−1^ and 6.7586 mg·L^−1^, respectively. A novel microbial metabolic pathway for 3-PBA was proposed on the basis of these metabolites. Inoculation of strain DG-02 resulted in a higher degradation rate on 3-PBA than that observed in the non-inoculated soil. Moreover, the degradation process followed the first-order kinetics, and the half-life (*t*_1/2_) for 3-PBA was greatly reduced as compared to the non-inoculated control. This study highlights an important potential application of strain DG-02 for the in situ bioremediation of 3-PBA contaminated environments.

## Introduction

3-Phenoxybenzoic acid (3-PBA) is a common primary metabolite of the synthetic pyrethroid insecticides (SPs) that have been extensively used in agriculture and urban areas because of their high efficiency and low mammalian toxicity [1]. In addition, it is also the important chemical raw material widely used in the manufacture of SPs and dyes [2]. A consequence of the increased availability, usage and broad-spectrum applicability of 3-PBA is its widespread exposure among the general population. Recent evidences suggest that 3-PBA is the most frequently detected xenobiotic in human urine [3]–[11]. Moreover, it also possesses endocrine disrupting activity [12]–[14], and adversely affects reproductive function and sexual development as well as the immune system in men [15]–[20]. As the general metabolic product of SPs, 3-PBA is considered far more potent as endocrine disruptor than the parent molecules [12], [14].

In the natural environment, 3-PBA is persistent and refractory to degradation. The half-life in soil is reported ranging from 120 to 180 days depending on soil type, climate and other conditions [21], [22]. Moreover, 3-PBA can enter into the aqueous phase, but it tends to be absorbed to the sediment, which in turn increases its half-life in soil [12]. The accumulation and widespread use of 3-PBA have resulted in its ubiquitous presence as a contaminant in urban sediments and surface water thus should be of great environmental and public health concern [2], [23], [24].

Effective measures for disposal of this toxic chemical are critically needed to ensure that human and environmental health will not be compromised by the continued use of 3-PBA. Bioremediation involving the use of living microorganisms in the removal of pollutants is the most promising, environmentally friendly, relatively efficient, and cost-effective process [25]. There is growing body of evidence that environmental hazardous materials could be successfully eliminated by diverse microorganisms belonging to different taxonomic groups [26]–[30]. However, limited reports have far been available in the literature regarding the microbial degradation of 3-PBA by a pure bacterial culture. A possible reason is attributed to its toxicity. Previous studies demonstrated that 3-PBA was refractory to microbial degradation and enhanced degradation could not occur in the pure culture [21], [31]. Although several bacterial strains have been reported to degrade low concentrations of 3-PBA [21], [22], [32], yet no report on mineralization of this compound is available. Halden et al. [21] reported firstly that *Pseudomonas pseudoalcaligenes* strain POB310 was able to utilize 3-PBA as a growth substrate in soil. But degradation of 3-PBA in soil by strain POB310 was incomplete, and bacterial densities decreased even under the most favorable conditions (100 mg·L^−1^ of 3-PBA, supplementation with P and N, and soil water-holding capacity of 90%). *Stenotrophomonas* sp. strain ZS-S-01 seems to be the most efficient strain, since it degraded 250 mg·L^−1^ of 3-PBA within 14 days with a degradation rate of 82.9% [22]. Furthermore, to the best of our knowledge, the microbial metabolic pathway of 3-PBA has not been investigated so far and remains unclear.

The objective of this study was to screen isolates that were capable of effectively degrading 3-PBA. Moreover, the metabolic pathway was first proposed and the potential for bioremediation of 3-PBA contaminated soils was also evaluated.

## Materials and Methods

### Chemicals and Media

Standards of 3-PBA (98% purity) and 3-phenoxybenzaldehyde (98% purity) were obtained from Sigma-Aldrich, USA. 3-Phenoxybenzyl alcohol standard (99% purity) was purchased from Dr.Ehrenstorfer GmbH, Germany. Chromatographic-grade acetonitrile and methanol were purchased from Burdic & Jackson, USA. All other chemicals and reagents used were of pure analytical-grade and available commercially. Stock solutions of different pyrethroid metabolites (10 g/L) were prepared with various solvents (including methanol, acetonitrile, and acetone), sterilized by membrane filtration (0.45 µm), and stored in dark bottles at 4°C before use.

The Luria-Bertani (LB) medium containing 10.0 g of tryptone, 5.0 g of yeast extract, and 10.0 g of NaCl per liter of water, and the mineral salt medium (MSM) containing 2.0 g of (NH_4_)_2_SO_4_, 0.2 g of MgSO_4_·7H_2_O, 0.01 g of CaCl_2_·2H_2_O, 0.001 g of FeSO_4_·7H_2_O, 1.5 g of Na_2_HPO_4_·12H_2_O, and 1.5 g of KH_2_PO_4_ per liter of water were used in this study. Both media were adjusted to a final pH value of 7.5, and sterilized at 121°C for 20 min separately.

### Enrichment and Isolation of the 3-PBA-degrading Bacteria

To isolate 3-PBA-degrading bacteria, an enrichment culture technique was performed as described previously [33]. Soil samples used as initial inoculants for enrichment were collected from aerobic pyrethroid-manufacturing wastewater treatment system located in Guangdong Province, China, which had produced SPs for over 10 years. Two grams of soil sample was transferred into a 250-mL Erlenmeyer flask containing 50 mL MSM with the addition of 50 mg·L^−1^ 3-PBA as the sole carbon source and incubated at 30°C for 7 days in a rotary shaker at 150 rpm. Five milliliters of the enrichment culture was transferred into 50 mL fresh enrichment medium containing 100 mg·L^−1^ of 3-PBA and incubated for another 7 days. Three additional successive transfers were made into MSM containing 200, 300, and 500 mg·L^−1^ of 3-PBA. The final culture was serially diluted and spread on LB agar plates. Colonies with different morphologies that grew on the plates were picked and purified using the streaking method. The ability of isolates to degrade 3-PBA was determined by high performance liquid chromatography (HPLC) (Agilent 1100, USA) as described previously [34]. One pure isolate showing the highest degradation was selected for further study and designated DG-02.

### Identification and Characterization of Strain DG-02

The isolate was grown on LB agar plates for 3 days and its morphology was determined by light microscopy (BH-2 Olympus, Japan) and scanning electron microscopy (XL-30 ESEM, Philips Optoelectronics Co., Ltd, Holland). Colony morphology was observed after incubation on LB agar plates at 30°C at 1, 2, and 3 days. Physio-biochemical tests were carried out referred to Bergey’s Manual of Determinative Bacteriology [35]. Total genomic DNA was prepared by the method of Sambrook and Russell [36]. The 16S rRNA gene was amplified by polymerase chain reaction (PCR) with the universal primers B1 (5′-AGAGTTTGATCCTGGCTCAG-3′, *Escherichia coli* bases 8–27) and B4 (5′-ACGGHTACCTTGTTTACGACTT-3′, *E.coli* bases 1507-1492) [37]. Reaction conditions consisted of initial denaturation at 95°C for 5 min, followed by 32 cycles of denaturation at 94°C for 45 s, annealing at 50°C for 45 s, and extension at 72°C for 1.25 min, with the last cycle followed by a ten-minute extension at 72°C. The PCR products were purified and ligated to the linearized vector pMD20-T (TaKaRa Biotechnology Co. Ltd., China), and transformed into competent *E.coli* DH5α cells. Sequencing of the cloned insert was performed by Shanghai Invitrogen Technology Co. Ltd., China. The resulting sequence was compared with the genes available in the GenBank nucleotide library by a BLAST search through the National Center for Biotechnology Information (NCBI) internet site. Multiple alignments of 16S rDNA were carried out using ClustalX 1.8.1 with default settings, and phylogenetic analysis was performed using MEGA 4.0 software. An unrooted tree was built following the neighbor-joining method [38]. Dataset was bootstrapped 1000 times.

### Growth and Degradation Assays

Strain DG-02 was cultured in LB liquid medium for about 24 h, harvested by centrifugation (4000×*g*, 5 min) and washed twice with 0.9% sterile normal saline. After the cell density had been adjusted to OD_600_ = 0.6, one percent of this suspension (1.0×10^8^ CFU·mL^−1^) was inoculated into 50 mL of MSM containing 50 mg·L^−1^ 3-PBA as the sole carbon source in a 250-mL Erlenmeyer flask, unless otherwise mentioned. Growth experiments were also performed in MSM supplemented with 1% (*w*/*v*) glucose [26]. Triplicate cultures were grown at 30°C and 150 rpm on a rotary shaker. Non-inoculated medium served as control. Samples (30-mL) were collected periodically from the cultures to measure the optical density (OD) value at 600 nm and to determine the 3-PBA concentration by HPLC according to the method of Xie et al. [24].

Degradation experiments with different initial concentrations of 3-PBA (25–500 mg·L^−1^) and with other diaryl ether compounds including 3-phenoxybenzaldehyde and 3-phenoxybenzyl (both 50 mg·L^−1^) by the isolate were also carried out in MSM supplemented with 1% glucose for 3 days at the optimum conditions.

### Optimization of 3-PBA-degrading Conditions by Strain DG-02

The culture conditions favoring 3-PBA degradation by strain DG-02 was investigated by using response surface methodology (RSM) [39]. The significant factors and their ranges that were selected as independent variables were temperature (20–40°C), pH (5.5–9.5), and inoculum (OD_600_ = 0.1–1.0). A three-level (−1, 0, 1) Box-Behnken design consisting of 15 experimental runs with three replicates at the center point was generated by statistic analysis system (SAS) software (Version 9.0). The symbols and levels of the three independent variables are presented in Table 1. The dependent variable was the degradation of 50 mg·L^−1^ 3-PBA in MSM supplemented with 1% glucose by strain DG-02 by day 2. Randomised block design was conducted in order to minimize the effects of unexplained variability in the observed response because of extraneous factors. The data were processed by using the response surface regression procedure of the SAS software to fit the following quadratic polynomial equation (Eq.(1)).

**Table 1:** Box-Behnken experimental design matrix and the response of dependent variable for 3-PBA degradation.

				Response
Run	*X*_1_ a	*X*_2_ b	*X*_3_ c	3-PBA degradation (%)*Y*_1_
1	−1	−1	0	46.2±1.1n
2	−1	1	0	49.1±1.8m
3	1	−1	0	48.8±2.2m
4	1	1	0	56.5±1.9l
5	0	−1	−1	62.3±1.2k
6	0	−1	1	65.5±1.6j
7	0	1	−1	75.2±1.7g
8	0	1	1	78.5±0.9e
9	−1	0	−1	70.9±0.7i
10	1	0	−1	77.1±0.8f
11	−1	0	1	74.3±0.6h
12	1	0	1	84.3±1.7d
13	0	0	0	90.5±0.9c
14	0	0	0	91.7±0.3b
15	0	0	0	93.4±0.7a

a refers to temperature: −1 (20°C), 0 (30°C), +1 (40°C);

b refers to pH: −1 (5.5), 0 (7.5), +1 (9.5);

c refers to inoculum: −1 (OD_600nm_ = 0.2), 0 (OD_600nm_ = 0.6), +1 (OD_600nm_ = 1.0). The data presented are means of three replicates with standard deviation, which is within 5% of the mean. Different letters indicate significant differences (*P*<0.05, LSD test).



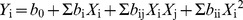
(1)where *Y*_i_ is the predicted response, *X*_i_ and *X*_j_ are variables, *b*_0_ is the constant, *b*_i_ is the linear coefficient, *b*_ij_ is the interaction coefficient, and *b*_ii_ is the quadratic coefficient.

### Identification of Metabolites during 3-PBA Degradation

The metabolic products of 3-PBA in cell-free filtrates of the bacterial cultures grown in MSM containing 50 mg·L^−1^ of 3-PBA were identified by gas chromatopraphy-mass spectrometry (GC-MS) (Agilent 6890N/5975, USA) as described previously [40]. The cell-free filtrates were collected at 24, 48, and 72 h, respectively. The metabolites confirmed on the basis of mass spectrum were matched with authentic standard compounds from the National Institute of Standards and Technology (NIST, USA) library database.

### Biodegradation of 3-PBA in Soil

A composite soil sample taken from the top layer (0–20 cm) at grass-covered field located in Guangzhou, Southern China, was used in this experiment. The sampling places have not been used for agricultural purposes during the past 5 years. The soil has never been treated with 3-PBA, pesticides, as well as organic and inorganic fertilizers. The soil sample was air dried and sieved to 2 mm prior to use. The physicochemical properties of the soil were (grams per kilogram of dry weight): organic matter, 10.5; total N, 0.5; total P, 0.4; total K, 18.2; and pH, 6.9. The soil has a sandy loam texture (sand 65.0%, silt 28.0%, clay 7.0%).

To evaluate the potential for bioremediation of 3-PBA polluted soils by strain DG-02, 250 g of soil was placed in 500 mL-Erlenmeyer flask and the water contents were adjusted to approximately 40% of water-holding capacity by the addition of sterile deionised water. All soil samples were incubated in a darked thermostatic chamber maintained at 30±1°C. Throughout the incubation period, the sterile deionised water was added to the soil treatments to compensate for any water losses exceeding 5% of the initial amount added [41]. 3-PBA was added to a final concentration of 50 mg·kg^−1^ of soil in methanol solution. After mixing and solvent evaporation the bacterial suspension was introduced into the soils (in triplicate) by drip irrigation to give a final concentration of 1.0×10^6^ CFU·g^−1^ soil. The inoculum was thoroughly mixed under sterile conditions. Additionally, sterilized soil (autoclaving at 121°C for 1 h) was also used to compare the removal efficiency of 3-PBA in the soil. Triplicate samples of non-inoculated soils were kept as control. Soil treatments (20 g) were periodically removed aseptically for chemical analysis to determine 3-PBA concentrations and to detect the metabolic products from the degradation of 3-PBA.

### Data Analysis

The substrate inhibition model (Eq.(2)) was applied to describe the specific degradation rate (*q*) at different initial concentrations of 3-PBA.
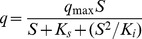
(2)where *q*_max_ is the maximum specific 3-PBA degradation rate (h^−1^), *K*_i_ is the substrate inhibition constant (mg·L^−1^), *K*_s_ is the half-saturation constant (mg·L^−1^), *S* is the inhibitor concentration (mg·L^−1^), and *S*_m_ is the critical inhibitor concentration of the substrate which decreases degradation.

The first-order kinetic model (Eq.(3)) was used to describe the rate of total 3-PBA reduction in soil.
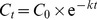
(3)where *C*_0_ is the initial concentration of 3-PBA at time zero, *C*_t_ is the concentration of 3-PBA at time *t*, *k* and *t* are the degradation rate constant (day^−1^) and degradation period in days, respectively.

The algorithm as expressed in Eq.(4) was used to determine the theoretical half-life (*t*_1/2_) values of 3-PBA in soil.
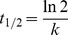
(4)where ln2 is the logarithmic function, *k* is the rate constant (day^−1^).

Results were also assessed by analysis of variance (ANOVA) and statistical analyses were performed on three replicates of data obtained from each treatment. The significance (*P*<0.05) of differences was evaluated by post hoc comparison of means using the least significant differences (LSD) test using SAS software packages. The data obtained for 3-PBA degradation in medium and 3-PBA degradation products were treated statistically by one-way ANOVA, considering the effect of medium type, while the results concerning the disappearance of 3-PBA in soil were analysed by two-way ANOVA, considering the effects of time and soil type. For 3-PBA optimum degradation conditions, the data were treated statistically by three-way ANOVA, considering the effects of temperature, pH, and inoculum.

### Ethics Statement

No specific permits were required for the described field studies. No specific permissions were required for these locations. We confirm that the location is not privately-owned or protected in any way. We confirm that the field studies did not involve endangered or protected species.

## Results

### Isolation and Characterization of Strain DG-02

After five rounds of transfer, a total of 7 pure isolates with unique morphologies were obtained from the enrichment culture. One strain, designated DG-02, was selected for further study due to its broad-spectrum substrate specificity and relatively high degradation rate for various diaryl ether compounds. This strain was capable of utilizing 3-PBA as the sole carbon source and degraded 95.6% of 50 mg·L^−1^ 3-PBA in less than 72 h.

**Figure 1:**
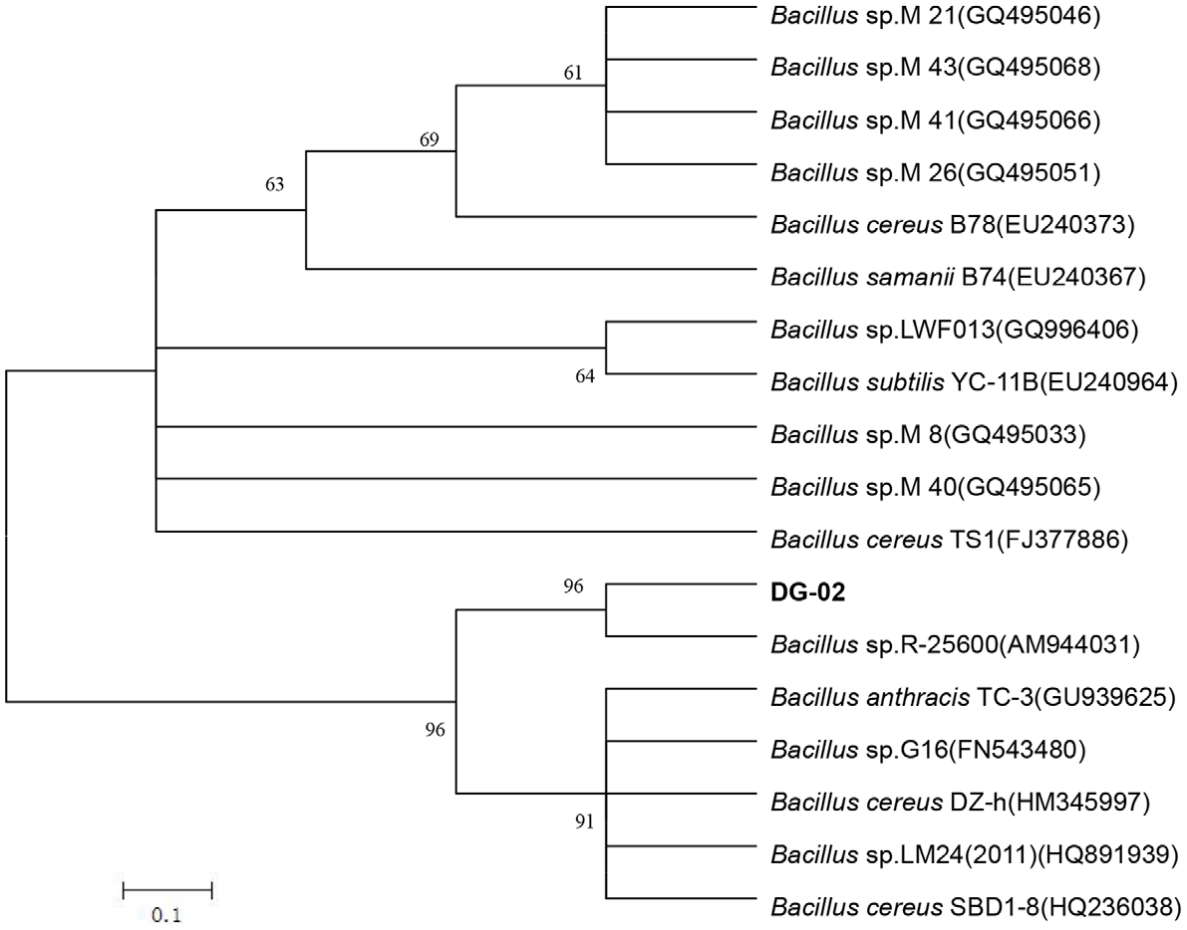
Phylogenetic tree based on the 16S rRNA sequences of strain DG-02 and related strains. Numbers in parentheses represent the sequences accession number in GenBank. Numbers at the nodes indicate bootstrap values from the neighborhood-joining analysis of 1,000 resampled data sets. Bar represents sequence divergence.

Strain DG-02 was a Gram-positive, obligately aerobic, rod-shaped bacterium with dimensions of 2.0 to 3.0 µm in length and 0.4 to 0.6 µm in width (Fig. S1). Colonies grown on LB agar plates were ivory, opaque, and circular with convex elevation entire margin. It was positive in tests or reactions such as catalase, amylohydrolysis, gelatin liquefaction, voges-proskauer (V-P), nitrate reduction, hydrogen sulfide production, indol reaction, and utilization of glucose and citrate. It was negative in utilization of arabinose, xylitol, and mannitol (Table 2). The DNA G+C content is 53.3 mol %. Phylogenetic analysis of the 16S rRNA gene sequences revealed that strain DG-02 grouped among *Bacillus* species and formed a subclade with *Bacillus* sp.R25600 with a high bootstrap value of 100% (Fig. 1). Thus, strain DG-02 belongs to the *Bacillus* group of bacteria based on the morphology, physio-biochemical characteristics, and 16S rRNA gene analysis. The 16S rRNA gene sequences with 1,515 bp were deposited at the GenBank under the accession number JN700990.

**Table 2:** Morphological and physio-biochemical characteristics of *Bacillus* sp. strain DG-02.

Characteristics	Results	Characteristics	Results
Colonies	Ivory, opaque, and circular with convex elevation entire margin	Cells	Rod-shaped, 2.0–3.0 µm in length, and 0.4–0.6 µm in width
Gram-staining	+	Catalase	+
Amylohydrolysis	+	Gelatin liquefaction	+
Hydrogen sulfide production	+	Nitrate reduction	+
Voges-Proskauer (V–P)	+	Indol reaction	+
Glucose	+	Citrate	+
Arabinose	−	Xylitol	−
Mannitol	−		

+, tested positive/utilized as substrate;

−, tested negative/not utilized as substrate.

### Utilization of 3-PBA for Growth by Strain DG-02

**Figure 2:**
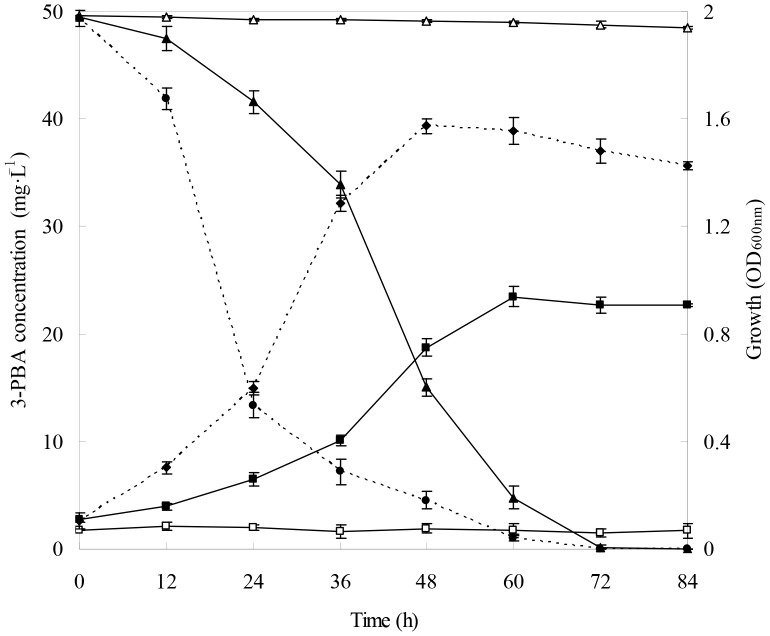
Degradation and utilization of 3-PBA during growth of strain DG-02. ▴, degradation kinetics in MSM supplemented with 3-PBA as the sole carbon source; •, degradation kinetics in MSM supplemented with 1% glucose as the additional source of carbon; △, non-inoculated control (degradation); ▪, cell growth in MSM supplemented with 3-PBA as the sole carbon source; ♦, cell growth in MSM supplemented with 1% glucose as the additional source of carbon; □; non-inoculated control (growth). Error bars represent the standard deviation of three replicates.

As shown in Fig. 2, strain DG-02 utilized 3-PBA as the sole carbon source for growth in MSM. Within 60 h, more than 90% of the 50 mg·L^−1^ 3-PBA initially added to the medium was degraded by strain DG-02. No significant change in 3-PBA concentration was observed in non-inoculated medium. 3-PBA degradation was associated with a concomitant increase of cell density, the OD_600_ value of the cell culture rose from 0.1 to 0.9. In the presence of 1% glucose, the growth was strongly stimulated in MSM whereas OD_600_ value of the cell culture was significantly increased (*P*<0.05) from 0.1 to 1.6 (Fig. 2). Meanwhile, disappearance rate of 3-PBA in MSM supplemented with glucose was significantly higher (*P*<0.05) and reached 91.0% within 48 h; while in the same period only 69.6% 3-PBA was degraded in MSM in the absence of other carbon source. Moreover, complete degradation was observed at the end of reaction. Significantly, addition of 1% glucose accelerated the degradation of 3-PBA by this strain.

### Optimization of the 3-PBA-degrading Conditions by Strain DG-02

Response surface methodology (RSM) based on the Box-Behnken design was applied to determine the main and interactive effects of significant variables including temperature (*X*_1_), pH (*X*_2_), and inoculum (*X*_3_) in this experiment. The experimental design matrix and the response of dependent variable for 3-PBA degradation are given in Table 1. Subsequently, the data from Table 1 were analyzed by response surface regression procedure of SAS software package, and the results of the quadratic polynomial model fitting in the term of analysis of variance (ANOVA) were presented in Table 3. A quadratic polynomial function was fit to the experimental values, resulting in the following regression equation (Eq.(5)):
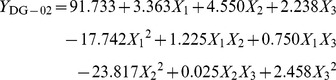
(5)where *Y*_DG-02_ is the predicted 3-PBA degradation (%) by strain DG-02; *X_1_*, *X_2_*, and *X_3_* are the coded values for the temperature, pH, and inoculum, respectively.

**Table 3:** Analysis of variance (ANOVA) for the fitted quadratic polynomial model for 3-PBA degradation.

Source	3-PBA degradation				
	DF^a^	SS^b^	MS^c^	*F* Value	*P* Level^d^
*X*_1_	1	90.5	90.5	10.4	0.0233
*X*_2_	1	165.6	165.6	19.1	0.0072
*X*_3_	1	40.1	40.1	4.6	0.0845
*X*_1_ *X*_1_	1	1162.2	1162.2	133.8	<0.0001
*X*_1_ *X*_2_	1	6.0	6.0	0.7	0.4437
*X*_1_ *X*_3_	1	2.3	2.3	0.3	0.6325
*X*_2_ *X*_2_	1	2094.4	2094.4	241.1	<0.0001
*X*_2_ *X*_3_	1	0.003	0.003	0.0003	0.9871
*X*_3_ *X*_3_	1	22.3	22.3	2.6	0.1699
Model	9	3448.9	383.2	44.1	0.0003
Lack of fit	3	38.9	13.0	5.8	0.1509
Pure error	2	4.5	2.2		
Total	14	3492.3			

*R* ^2^ = 0.9876; coefficient of variation (CV) = 4.2%; Adj.*R* ^2^ = 0.9652.

a refers to degrees of freedom;

b refers to sum of sequences;

c refers to mean square;

d *P* Level less than 0.05 indicates the model terms are significant.

The statistical significance of Eq.(5) was also assessed by performing *F*-test and ANOVA (Table 3). The adequacy of the model was indicated by the determination coefficient (*R* ^2^ = 0.9876), which explained 98.76% of the response variability, suggesting that the predicted values of the model were in perfect agreement with the experimental values. The high value of the adjusted *R* ^2^ (0.9652) further supported the accuracy of the model. The lack of fit value was not significant (*P*>0.05), indicating that the equation was adequate for predicting 3-PBA degradation. The low coefficient of variation (CV = 4.2%) demonstrated that the model was precise and reliable. The developed exact model thus could be effectively used to predict and optimize the 3-PBA-degrading conditions within the limits of chosen factors.

**Figure 3:**
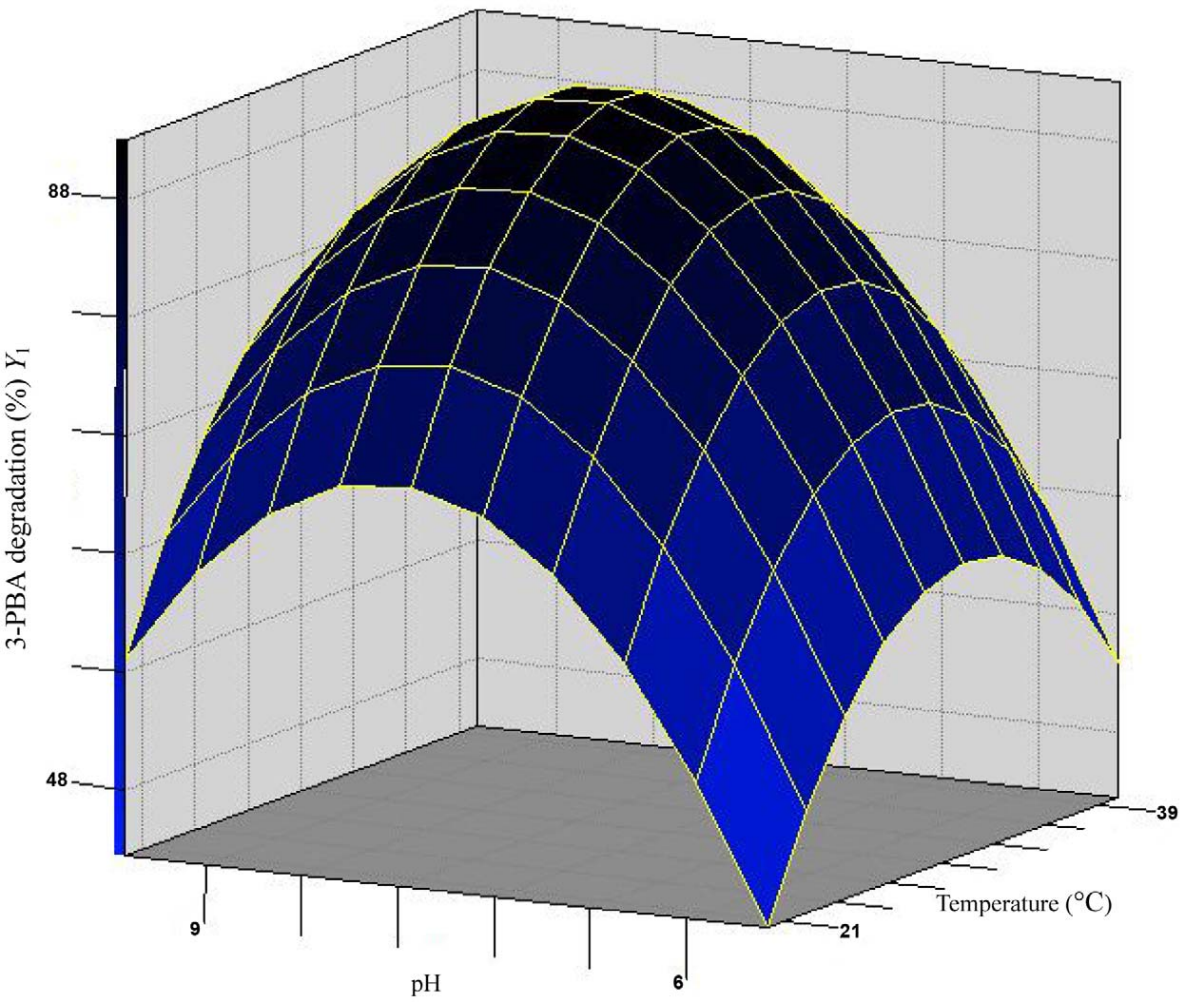
Response surface plot showing the effects of temperature and pH on 3-PBA degradation by strain DG-02 with inoculum at OD_600_ = 0.6.

The results of the regression parameter estimate revealed that linear and square terms of temperature (*X*_1_) and pH (*X*_2_) values showed significant effects (*P*<0.05) on the 3-PBA degradation by strain DG-02, but the linear term of inoculum (*X*_3_) and the interaction terms were insignificant (*P*>0.05). With the value of inoculum (the non-significant variable) fixed at OD_600_ = 0.6, a three-dimensional response surface was plotted to directly display the effects of temperature and pH on the 3-PBA degradation by strain DG-02 (Fig. 3). As shown in Fig. 3, the plot of 3-PBA biodegradation had a theoretical maximum value of 91.6% at the stationary point. At the stationary point, the optimum levels for the two variables of *X_1_* and *X_2_* were found to be 0.088 and 0.098 in terms of the coded units, that is, temperature 30.9°C and pH 7.7, respectively. So the optimum conditions for 3-PBA degradation by strain DG-02 were determined to be 30.9°C, pH 7.7, and inoculum at the OD_600_ of 0.6.

### Degradation of 3-PBA with Different Initial Concentrations by Strain DG-02

**Figure 4:**
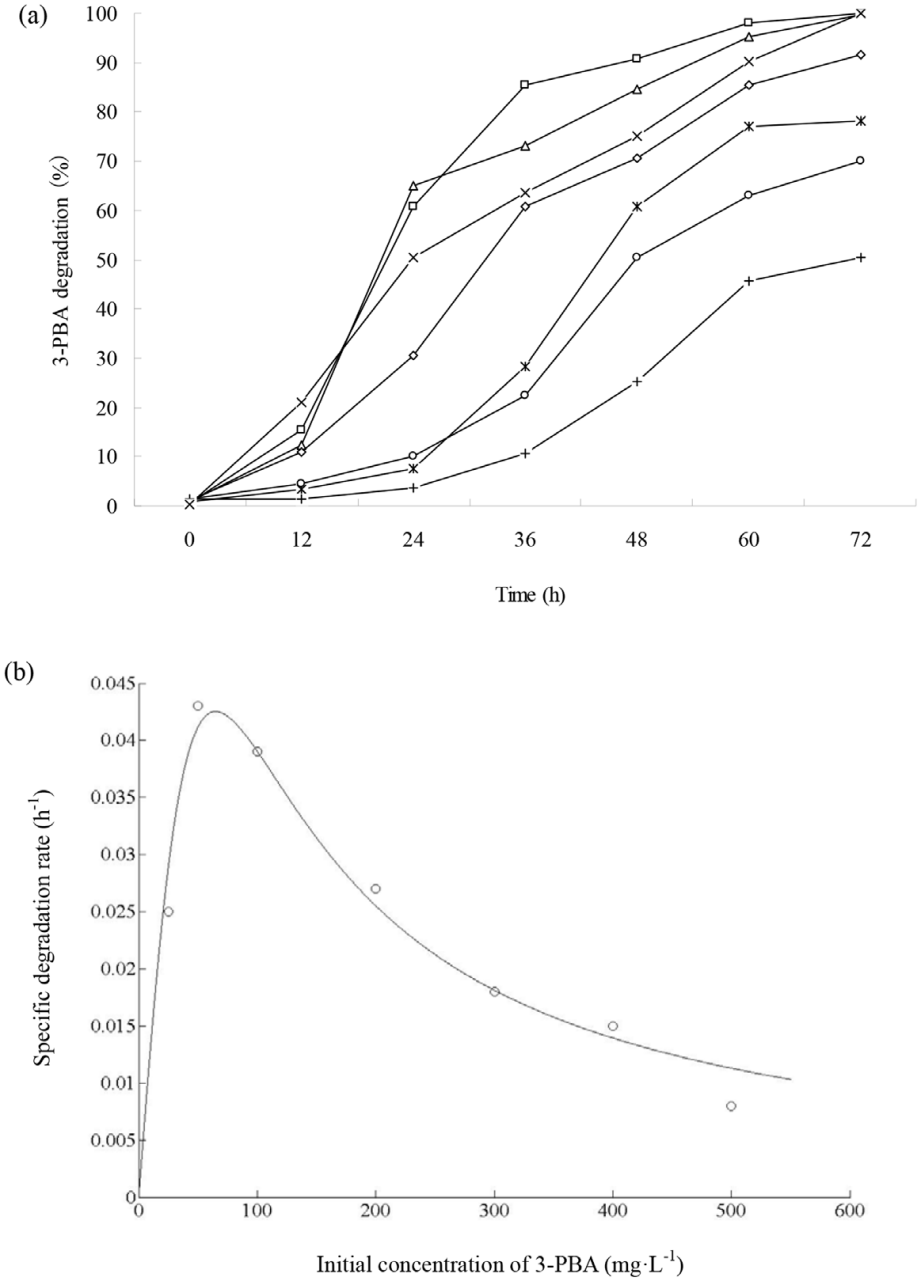
(a) Degradation of 3-PBA with different initial concentrations by strain DG-02. △, 25 mg·L^−1^; □, 50 mg·L^−1^; ×, 100 mg·L^−1^; ◊, 200 mg·L^−1^; *, 300 mg·L^−1^; ○, 400 mg·L^−1^; +, 500 mg·L^−1^. (b) Relationship between specific degradation rate and initial 3-PBA concentration.

Fig. 4a shows the degradation kinetics of 3-PBA with different initial concentrations by strain DG-02. Strain DG-02 utilized and grew on 3-PBA up to the concentration of 500 mg·L^−1^, and the lag phase was extended at higher 3-PBA concentration. The degradation rates reached 100% within 72 h at low initial concentrations of 25, 50, and 100 mg·L^−1^. However at higher initial concentrations of 200, 300, 400, and 500 mg·L^−1^, the degradation rates only reached 91.7%, 78.1%, 70.0%, and 50.5% after 72 h of incubation, respectively.

The decrease in the specific 3-PBA degradation rate with an increase in the initial 3-PBA concentration suggested that 3-PBA may act as an inhibitor to strain DG-02. Thus, the substrate inhibition model (Eq.(2)) was used to fit the specific degradation rate (*q*) at different initial concentrations. The relationship between *q* and initial 3-PBA concentration is shown in Fig. 4b. Through the non-linear regression analysis by matrix laboratory (MATLAB) software (Version 7.8), the kinetic parameters *q_max_*, *K*_s_ and *K*_i_ were estimated to be 0.8615 h^−1^, 626.7842 mg·L^−1^, and 6.7586 mg·L^−1^, respectively. The critical inhibitor concentration (*S*_m_) was determined to be 65.0858 mg·L^−1^. The value of *R* ^2^ was 0.9702 indicating that the experimental data was well correlated with the model. As shown in Fig. 4b, when the initial concentrations of 3-PBA were lower than 65 mg·L^−1^, *q* gradually increased. At higher concentrations, inhibition by 3-PBA became prominent. It is a fact that 3-PBA displays the inhibitory effect at high concentration.

### Degradation of Various Diaryl ether Compounds by Strain DG-02

The ability of strain DG-02 to degrade a variety of diaryl ether compounds was investigated. The results showed that the strain was capable of degrading all the compounds tested. 3-PBA was the most preferred substrate, with a degradation rate of 100% within 72 h. 3-Phenoxybenzaldehyde was degraded slightly slower than 3-PBA, and 96.5% of the added substrate was degraded. However, only a relatively small fraction of 3-phenoxybenzyl alcohol was degraded, with a degradation rate of 56.2%. Abiotic degradation was negligible in the non-inoculated controls throughout all studies.

### Metabolites of 3-PBA Degradation by Strain DG-02

The degradation products of 3-PBA in cell-free filtrates were isolated and identified by GC-MS. The GC-MS analysis of the sample showed five peaks at retention times (RT) of 17.622, 15.903, 19.426, 3.718, and 11.039 min representing metabolites A, B, C, D, and E, respectively, as summarized in Table 4.

**Table 4:** Chromatographic properties of metabolites of 3-PBA degraded by strain DG-02.

Code	Retention time (min)	*m*/*z*	Compounds
A	17.622	271	3-PBA
B	15.903	480	3-(2-Hydroxyphenoxy) benzoic acid
C	19.426	499	Protocatechuate
D	3.718	281	Phenol
F	11.309	259	3,4-Dimethoxy phenol

Each of the five peaks was confirmed on the basis of its mass spectra and the NIST library identification program. Compound A had the same retention time as the 3-PBA standard (RT = 17.622 min). The mass spectral data also indicated that compound A was 3-PBA (Fig. S2a). This peak disappeared concomitantly with formation of another four new peaks corresponding to those of authentic 3-(2-hydroxyphenoxy) benzoic acid, protocatechuate, phenol, and 3,4-dihydroxy phenol (Fig. S2b,c,d,e) based on the characteristic fragment ion peaks and molecular ion (*m*/*z*). However, these peaks were transient and they disappeared finally. Eventually, no persistent accumulative metabolite was detectable by GC-MS after dissipation of the parent compound.

**Figure 5:**
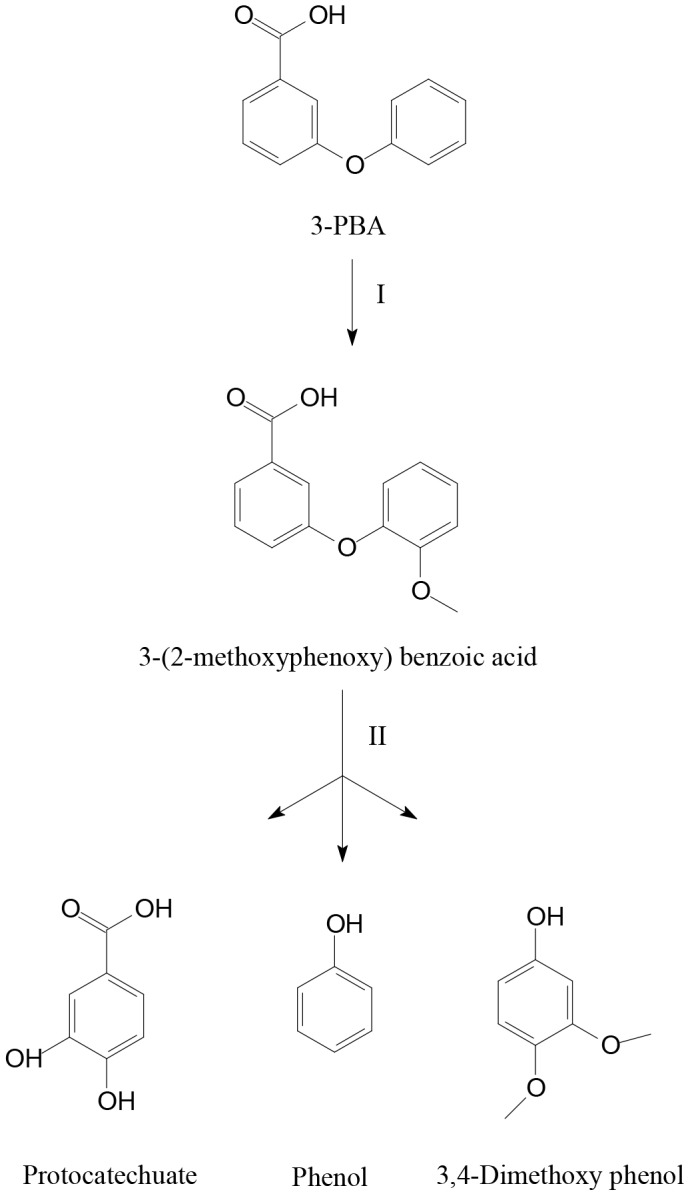
The proposed pathway for the 3-PBA degradation by strain DG-02. I, oxidization; II, phenyl ether cleavage.

According to the metabolic products formed, a novel degradation pathway for 3-PBA by strain DG-02 was proposed (Fig. 5). The parent 3-PBA [A] was first metabolized by oxidization to yield 3-(2-hydroxyphenoxy) benzoic acid [B]. Subsequently, the intermediate was further transformed by cleavage of the diaryl ether, resulting in formation of protocatechuate [C], phenol [D] and 3,4-dihydroxy phenol [E]. This is the first report of the pathway of 3-PBA degradation by oxidization and cleavage of the diaryl ether in a microorganism.

### Degradation of 3-PBA in Different Contaminated Soils by Strain DG-02

**Figure 6:**
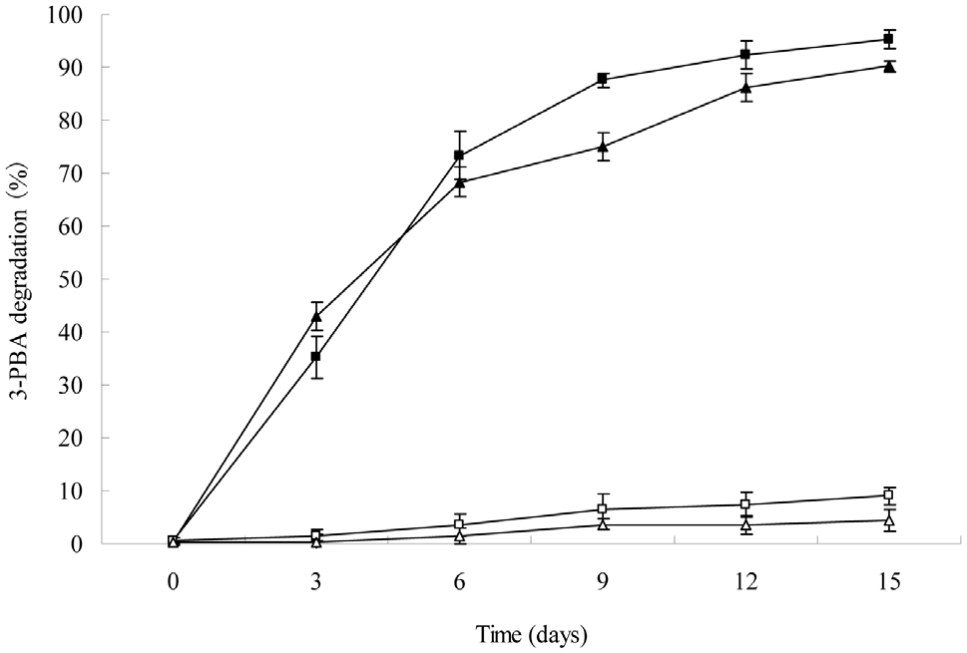
Degradation of 3-PBA in different contaminated soils by strain DG-02. △, non-inoculated control (sterilized soil); □, non-inoculated control with indigenous microbial community (non-sterilized soil); ▴, sterilized soil introduced with strain DG-02; ▪, non-sterilized soil introduced with strain DG-02. Error bars represent the standard deviation of three replicates.

The effects on the degradation of 3-PBA in different contaminated soils by strain DG-02 were determined in laboratory for 15 days (Fig. 6). In the non-sterilized soil introduced with strain DG-02 (1.0×10^6^ CFU·g^−1^ soil), rapid and accelerated degradation was observed at the beginning of incubation, apparently there was no lag period and 95.2% of the 50 mg·kg^−1^ 3-PBA initially added to the soil was eliminated at the end of experiment; while in the non-inoculated control with indigenous soil microorganisms, the total 3-PBA content decreased only by 9.0%. In the case of sterilized soil inoculated with strain DG-02, similar enhanced degradation was observed and 90.2% of added 3-PBA was removed from the soil within 15 days. In contrast, more than 95% of the added 3-PBA remained in the sterilized soil that was not inoculated, and only 4.5% of the added 3-PBA was degraded during the experiment. Without any inoculum, there was a lag period of approximately 3 days before 3-PBA degradation in the soils; whereas apparently no lag phase was observed with the presence of strain DG-02. Moreover, trace amounts of 3-PBA metabolites protocatechuate, phenol, and 3,4-dimethoxy phenol were also detected in soil inoculated with strain DG-02, but no metabolites were found in the non-inoculated soil (data not shown). Incubation with strain DG-02 thus resulted in greater 3-PBA degradation as compared to that in the non-inoculated control. Additionally, the disappearance rate of 3-PBA in the non-sterilized soil was higher (*P*<0.05) than that in the sterilized soil, suggesting that the indigenous soil microorganisms promoted the ability of strain DG-02 to eliminate 3-PBA.

The kinetic parameters confirmed the effects on degradation of 3-PBA in various soils treated by strain DG-02 (Table 5). In these studies, the degradation rate and the 3-PBA concentration were in direct proportion, and the degradation process followed the first-order kinetics with a *R* ^2^ ranging from 0.9633 to 0.9932, indicating that the experimental data were well correlated with the model. The kinetic constants (*k*) for 3-PBA in the non-sterilized and sterilized soils with strain DG-02 were 0.2014 and 0.1694 day^−1^, respectively; whereas without the strain DG-02 the *k* were 0.0068 and 0.0037 day^−1^, respectively. Half-lives (*t*_1/2_) for 3-PBA in the non-sterilized and sterilized soils with strain DG-02, calculated from the linear equation obtained from the regression between ln(*C*_t_/*C*_0_) of the chemical data and time were 3.4 and 4.1 days, respectively. In contrast, the *t*_1/2_ for 3-PBA in the non-inoculated soil treatments were 101.9 and 187.3 days, respectively. These results demonstrated that inoculation with strain DG-02 resulted in accelerated degradation in soil and higher 3-PBA degradation (*P*<0.05) was observed in the non-sterilized soil by a comparison of the level in the sterilized soil.

**Table 5:** Kinetic parameters of degradation of 3-PBA in different contaminated soils by strain DG-02.

Soil treatments	Regression equation	*k* (day^−1^)	*t*_1/2_ (days)	*R* ^2^
SS^a^ +3-PBA	*C_t_* = 50.0439e^−0.0037*t*^	0.0037	187.3	0.9691
nSS^b^ +3-PBA	*C_t_* = 50.4662e^−0.0068*t*^	0.0068	101.9	0.9633
SS+3-PBA+DG-02	*C_t_* = 49.1112e^−0.1694*t*^	0.1694	4.1	0.9932
nSS+3-PBA+DG-02	*C_t_* = 51.1561e^−0.2014*t*^	0.2014	3.4	0.9834

a refers to sterilized soil;

b refers to non-sterilized soil with indigenous soil microorganisms. Each figure in the table represents the mean of three replicates. All figures were significantly different at *P*<0.05.

## Discussion

Given the increased 3-PBA use around the world and its prevalent human exposure, effective techniques for disposal of the pollutant are critically needed. Microorganisms are not only cost-effective but also show good detoxification efficiency with contaminates and provide an environmentally friendly solution to the problem of 3-PBA pollution [42]. Unfortunately, attempts to isolate microorganisms in pure culture able to mineralize 3-PBA have usually failed, primarily due to the reason that this chemical is a recalcitrant substrate [21]. It has been suggested that the accumulated 3-PBA, which has antimicrobial property, prevents the proliferation of pyrethroid-degrading microorganisms [22], [32].

With the aim of obtaining efficient degradative microorganisms, in the last few years our laboratory has screened a range of contaminated soils for 3-PBA degradation activity. A bacterial strain, designated DG-02, was successfully isolated from the soil with a high efficiency in degrading 3-PBA. 16S rRNA gene sequencing and the physio-biochemical characteristics strongly suggested that strain DG-02 belongs to the *Bacillus* genus. As far as we know, there is no any information concerning the 3-PBA degradation by bacterial strains belonging to the *Bacillus* genus. Although *Bacillus* bacteria have been found to degrade a wide range of different xenobiotic aromatic compounds, including thiophanate-methyl [41], cypermethrin [43], chlorpyrifos [44], and 4-chloro-2-nitrophenol [45], our current results firstly describe the biodegradation of 3-PBA by this genus bacteria and definitely expand this list. It is thus becoming evident that *Bacillus* sp. is ubiquitous in the environment and possesses broad catabolic capabilities.

*Bacillus* sp. strain DG-02 was capable of degrading a variety of diaryl ether compounds, including 3-PBA, 3-phenoxybenzaldehyde, and 3-phenoxybenzyl alcohol. However, the degradation efficiencies were greatly different, suggesting that certain structural requirements must be met for a diaryl ether compound to be effectively metabolized by strain DG-02. 3-PBA was degraded faster than 3-phenoxybenzaldehyde, indicating that substitution of the acid group with an aldehyde group reduces the degradation efficiency. 3-Phenoxybenzyl alcohol was the most persistent, suggesting that replacement the acid group with an alcohol group hinders the 3-PBA hydrolase-substrate interaction in strain DG-02. Another possible reason for degradation reduction could be attributed to the fact that 3-phenoxybenzyl alcohol is more toxic and refractory to microbial degradation than 3-PBA [12], [17], [46]. The different capacities of the bacterium to metabolize these chemicals could have been due to a number of factors, including transport, enzyme specificity, or the molecular structure of the diaryl ether compounds. A similar observation has been reported in *Pseudomonas* sp. strain ET1 [23].

It was noteworthy that *Bacillus* sp. strain DG-02 degraded and utilized 3-PBA as a growth substrate in MSM (Fig. 2). Moreover, supplementation of 1% glucose in MSM greatly enhanced the transformation of 3-PBA, thus reflecting excellent environmental adaptation. This is an important feature of a pesticide-degrading microorganism to be employed for bioremediation of variable environments. However, this observation did not quite agree with Cycoń et al. [41] who found supplementation with additional carbon sources led to a lag period followed by enhanced degradation, since these strains preferred to utilize readily available carbon sources.

In the current study, the particular strain was found to efficiently degrade 3-PBA over a broad range of temperature (20–40°C) and pH (5.5–9.5), as shown in Table 1. This phenomenon gives pesticide-degrading microorganisms advantage in the environment, because they survival and utilize xenobiotics even exposed to adverse condition [47]. In addition, the optimum conditions for 3-PBA degradation by strain DG-02 were determined to be 30.9°C and pH 7.7 based on the response surface methodology (RSM). Under these conditions, strain DG-02 successfully remove the 50 mg·L^−1^ 3-PBA initially added to the medium within 72 h. It is interesting to note that the degradation was higher at the alkaline conditions. This might be because the higher pH increases the rate of 3-PBA transformation, whereas the acidic conditions may increase the 3-PBA stability and its resistance to chemical and microbial degradation, as described previously by Cycoń et al. [41] for thiophanate-methyl.

Another important feature which is worth mentioning is that this strain engaged in efficient degradation of 3-PBA up to the concentration, as high as 500 mg·L^−1^. In contrast to other reports on toxic effects of 3-PBA on different bacteria [21], [31], our results suggested that 3-PBA metabolism activity of *Bacillus* sp. strain DG-02 was not subject to complete catabolite repression by high 3-PBA concentration. However, the specific 3-PBA degradation rate slightly decreased with an increase in the initial 3-PBA concentration, and the critical inhibitor concentration (*S*_m_) was determined to be 65.0858 mg·L^−1^ (Fig. 4b).

On the basis of the results of metabolite identification, the metabolic pathway of 3-PBA was first proposed (Fig. 5). The transient accumulation of 3-(2-hydroxyphenoxy) benzoic acid indicated that the degradation of 3-PBA by *Bacillus* sp. strain DG-02 was initiated by oxidization (Fig. 5, step I). Moreover, small amounts of the metabolites protocatechuate, phenol, and 3,4-dimethoxy phenol followed by cleavage of the diaryl ether (Fig. 5, step II), were detected during the degradation of 3-PBA by strain DG-02. Since no abiotic degradation of 3-PBA to these products was observed in cell-free control, the metabolism of 3-PBA was apparently initiated by enzymatic reactions. This is the first evidence of a novel pathway of degradation of 3-PBA by oxidization and cleavage of the diaryl ether in a microorganism, which plays an important role in the 3-PBA biogeocycle.

In our studies, strain DG-02 showed a high survivability in the environment and maximal degradation activity on 3-PBA. As shown in Fig. 6, the introduced strain DG-02 quickly adapted to the environment and rapidly bioremediated 3-PBA contaminated soils during the first incubation period (3 days), apparently there was no lag phase. At the end of the experiment, 90.1% and 95.2% of the added 3-PBA (50 mg·kg^−1^) were removed from the sterilized and non-sterilized soils, respectively, and the *t*_1/2_ for 3-PBA was greatly reduced by 124.4 and 98.5 days as compared to the controls, respectively. Previous reports showed that the bacterial isolates introduced into the contaminated soil usually failed to degrade the pollutants due to the low activity of these bacteria caused by abiotic and biotic stresses, thus additional treatments were needed to enhance the degradation [44]. In this study, the bacterial isolate was found to efficiently degrade 3-PBA when inoculated into the soil, suggesting that strain DG-02 can be used directly for the elimination of 3-PBA in soil without any other further treatment. In addition, the removal efficiency of 3-PBA in the non-sterilized soil was significantly higher (*P*<0.05) than that in the sterilized soil, demonstrating that the indigenous soil microorganisms strongly stimulated the strain DG-02 to degrade 3-PBA. Enhancement of 3-PBA degradation could be explained by the fact that the introduced strain DG-02 and soil microorganisms may have a synergistic effect on metabolization of this recalcitrant, as described previously by Chen et al. [22], [30], with the same soil as used in our study. Moreover, analysis of 3-PBA degradation products in soil indicated that the degradation of 3-PBA by strain DG-02 was accompanied by transient accumulation of protocatechuate, phenol, and 3,4-dimethoxy phenol, but they were subsequently degraded rapidly and disappeared finally, which was in line with the 3-PBA degradation in liquid medium.

In conclusion, strain DG-02 isolated in the present study was found highly efficient in degrading 3-PBA in different contaminated soils and water resources, thus suggesting the isolate may be a promising candidate for bioremediation of 3-PBA contaminated environments. This is the first report about biodegradation of 3-PBA by a bacterial strain from the *Bacillus* genus. Moreover, the bacterium harbors the metabolic pathway for the detoxification of 3-PBA. This is the first evidence of a novel pathway of degradation of 3-PBA by oxidization and cleavage of the diaryl ether in a microorganism, which plays an important role in the 3-PBA biogeocycle.

## Supporting Information

Figure S1 **Morphological characteristics of strain DG-02 under scanning electron microscopy (15,000×).**
(TIF)

Figure S2 **GC-MS spectra of metabolites produced from 3-PBA degradation by strain DG-02.** a, 3-PBA; b, 3-(2-hydroxyphenoxy) benzoic acid; c, protocatechuate; d, phenol; e, 3,4-dimethoxy phenol.
(TIF)
