# Supplementary figures and images for: Degradation of 3-Phenoxybenzoic Acid by a Bacillus sp
Source: PLoS One. 2012 Nov 30;7(11):e50456. doi: 10.1371/journal.pone.0050456 (PMC3511583; doi:10.1371/journal.pone.0050456)

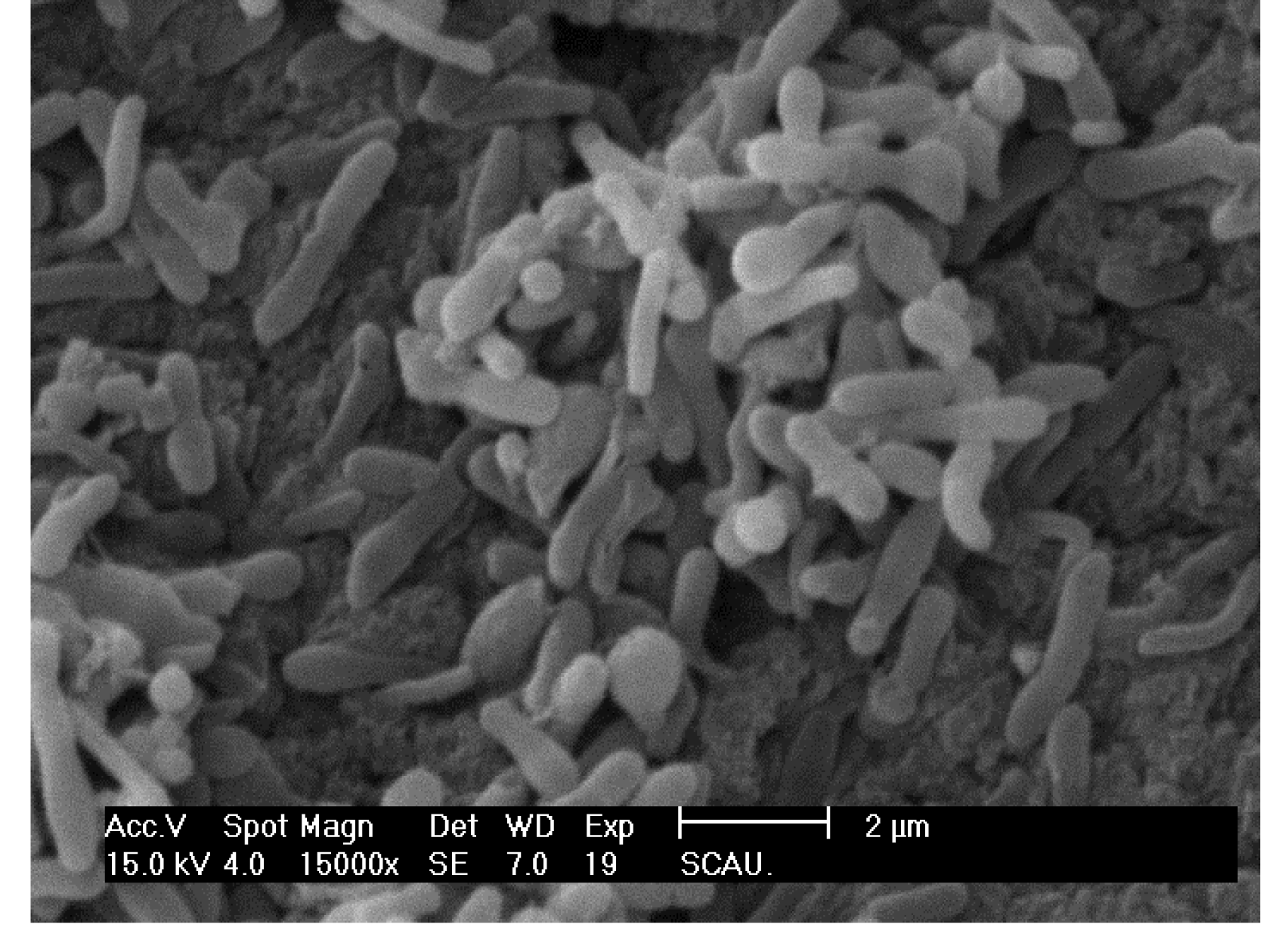

Supplement: Figure S1 — Morphological characteristics of strain DG-02 under scanning electron microscopy (15,000×). (TIF) [file pone.0050456.s001.tif]

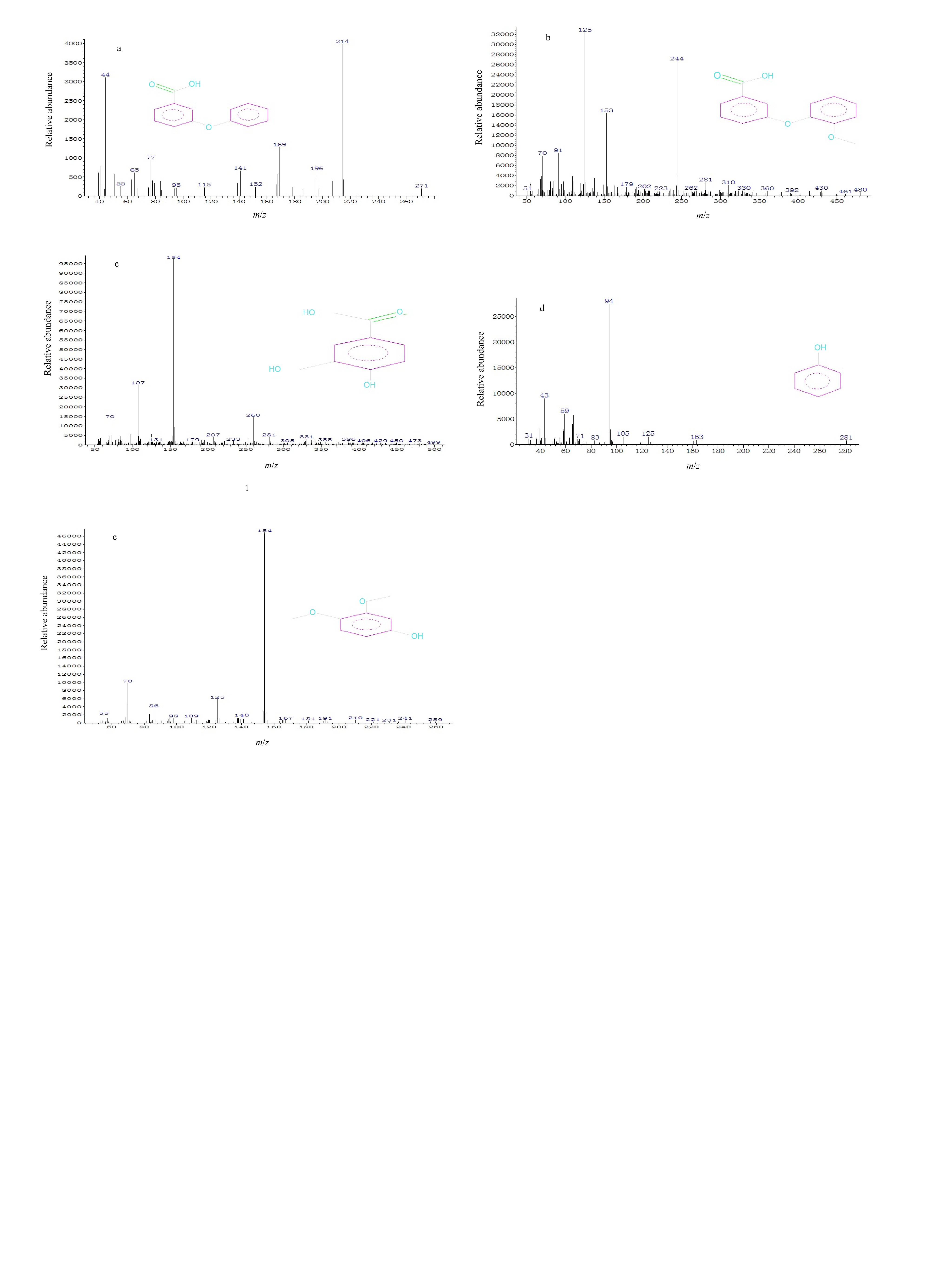

Supplement: Figure S2 — GC-MS spectra of metabolites produced from 3-PBA degradation by strain DG-02. a, 3-PBA; b, 3-(2-hydroxyphenoxy) benzoic acid; c, protocatechuate; d, phenol; e, 3,4-dimethoxy phenol. (TIF) [file pone.0050456.s002.tif]
